# Inhumation of Living Individuals Supposed to Be Dead

**Published:** 1846-10

**Authors:** 


*Inhumation of living Individuals supposed to be dead.*—According to an official statistical account, the number of premature interments, interrupted by fortuitous circumstances alone, have amounted, in France, since 1833, to 94. Of this number, 85 persons have awoke from lethargy themselves, at the moment when the funeral ceremonies were about to commence; 13 have been awaked under the excitement of means practised by the tenderness of the friends; 6 in consequence of the fall of the coffin in which they were confined; 9 owe their recovery to punctures made in the body on attaching the grave-clothes; 5 to the feeling of suffocation they experienced in the coffin; 19 to the accidental delay of the burial; 6 to voluntary delay, caused by doubts of death.—*Monthly Journal Medical Science, June*, 1846, *from Encyclographie Medical, April*, 1846.

